# A nation-wide retrospective epidemiological study of gastroenteropancreatic neuroendocrine neoplasms in china

**DOI:** 10.18632/oncotarget.17599

**Published:** 2017-05-03

**Authors:** Jin-Hu Fan, Yu-Qing Zhang, Su-Sheng Shi, Yuan-Jia Chen, Xing-Hua Yuan, Li-Ming Jiang, Shao-Ming Wang, Li Ma, Yu-Tong He, Chang-Yan Feng, Xi-Bin Sun, Qing Liu, Katrina Deloso, Yihebali Chi, You-Lin Qiao

**Affiliations:** ^1^ Department of Cancer Epidemiology, Cancer Hospital, Chinese Academy of Medical Sciences & Peking Union Medical College; ^2^ Department of Pathology, Cancer Hospital, Chinese Academy of Medical Sciences & Peking Union Medical College; ^3^ Department of Gastroenterology, Peking Union Medical College Hospital, Peking Union Medical College, Chinese Academy of Medical Sciences; ^4^ Department of Abdominal Surgery, Cancer Hospital, Chinese Academy of Medical Sciences & Peking Union Medical College; ^5^ Department of Radiology, Cancer Hospital, Chinese Academy of Medical Sciences & Peking Union Medical College; ^6^ Department of Epidemiology, Dalian Medical University; ^7^ Hebei Cancer Registry, the Fourth hospital of Hebei medical university; ^8^ Department of Nutrition, Chongqing Cancer Hospital & Institute & Cancer Center; ^9^ Department of Cancer Epidemiology, Henan Cancer Hospital/Institute; ^10^ Department of Cancer Prevention, Sun Yat-sen University Cancer Center; ^11^ Division of Biological Sciences, the University of Chicago; ^12^ Department of Medical Oncology, Cancer Hospital, Chinese Academy of Medical Sciences & Peking Union Medical College

**Keywords:** neuroendocrine tumors, digestive system, epidemiology, retrospective study multicenter study

## Abstract

**Background:**

Representative data on the gastroenteropancreatic neuroendocrine neoplasms (GEP-NENs) in Asian patients is rare, especially in China. This study aims to create a GEP-NENs profile of Chinese patients.

**Methods:**

This was a hospital-based, nation-wide, and multi-center 10-year (2001-2010) retrospective study which collected GEP-NEN patients’ information in tertiary referral hospitals. All 2010 inpatient GEP-NEN cases with confirmed pathology in the selected hospitals were included. The primary GEP-NEN sites were measured and the epidemiological and clinical information of each tumor site were compared.

**Results:**

The most common primary sites for GEP-NEN were the pancreas (31.5%) and rectum (29.6%), followed by the cardia (11.6%) and body (15.4%) of stomach. Small intestinal and colonic NENs took up a relatively small proportion of all patients. Pancreatic and rectal NENs, rather than cardiac and gastric body NENs, tended to be found in younger (*P*<0.001), female (*P*<0.001), urban (*P*<0.001) residents with a higher education level (P=0.032) and were also diagnosed at earlier stage (*P*<0.001) and lower grade (*P*<0.001). Surgery remained the primary treatment method in all groups.

**Conclusions:**

More studies on the commonality and heterogeneity of GEP-NENs are warranted to improve diagnosis efficiencies and treatment outcomes.

## INTRODUCTION

Neuroendocrine neoplasms (NENs) arise from cells throughout the diffuse endocrine system and are characterized by their ability to produce peptides that cause distinctive hormonal syndromes. NENs in the tubular digestive tract (including the pancreas) are known as gastroenteropancreatic neuroendocrine neoplasms (GEP-NENs).

Since the 1900′s, when Oberndorfer coined the name ‘karzinoide’ to describe submucosal neoplasms in small intestines [1], many nomenclatures systems have emerged. William and Sandler [2] classified all neuroendocrine neoplasms as foregut, midgut, or hindgut neoplasms according to their embryological origins, which covered the diverse features. The World Health Organization (WHO) provided a classification system in 2000 [3] based on a combination of pathological and clinical parameters, subsequently, the European Neuroendocrine Tumor Society (ENETS) [4, 5] and the American Joint International Cancer/ Union of International Cancer (AJCC/UICC) [6] proposed classification systems including TNM stage (the inherent biologic aggressiveness of the tumor), and grade (which refers to similarities between neoplastic cells and their non-neoplastic counterparts). Later classification systems, including WHO 2010, included both stage and grade systems. This lack of consensus on classification resulted in difficulty in investigating the epidemiology of GEP-NENs [7].

Several studies have revealed that the incidence of malignant neuroendocrine neoplasms (NENs) has been steadily increasing. The Surveillance, Epidemiology, and End Results (SEER) program [8] showed an increase from 1.09 new cases per 100,000 in 1973 to 5.25 per 100,000 in 2004, with 2.65 times as many GEP-NEN incidences in 2007 as there were in 1973 [9]. In the UK, gastrointestinal NEN (excluding pancreatic NENs) incidence increased by 4.8 and 3.8 times in males and females, respectively, from the 1970s to the years between 2000 and 2006 [10]. Furthermore, in the US, GEP-NEN incidence and predilection site differed between Caucasians, African Americans, and Asian/Pacific Islanders, suggesting a racial disparity in NENs [8]. Studies from Asian countries on this rare cancer have emerged recently and all revealed epidemiological inequalities with American and European countries [11–13]. Large-scale surveys are needed in mainland China to gather sufficient data on the features of this tumor. In this paper, we retrospectively collected epidemiological information and studied the current diagnosis and treatment of pathology-confirmed GEP-NEN cases in the past decade on a national scale.

## RESULTS

A total of 2,049 clinical records were retrieved from 23 hospitals. Eight cases were excluded because they involved tumors that had initially formed in the gallbladder and liver, and an additional 31 cases were excluded because the patients suffered from more than one type of cancer and the specimens in hospital were adenocarcinoma or squamous carcinoma. As a result, a final total of 2,010 patients were included, and of which 871 from specialized cancer hospitals and 1,139 came from general hospitals.

### Patients’ characteristics

Age range of the GEP-NEN patients was 8-89 years. Distribution of age did not comply with normal distribution (P=0.002), and the median age was 53.0 years (inter-quartile range: 20.0 years). The peak age group at diagnosis was 50-60 years. The sex ratio (men/women) was 1.4/1 (1169/841); 178 (35.4%) of 503 cases lacked a middle school education; 93.9% (1877 in 1999) of patients were married; and 1300 (68.1%) of 1909 cases with household registration information were urban residents (the remaining 609 were rural residents).

**Figure 1:**
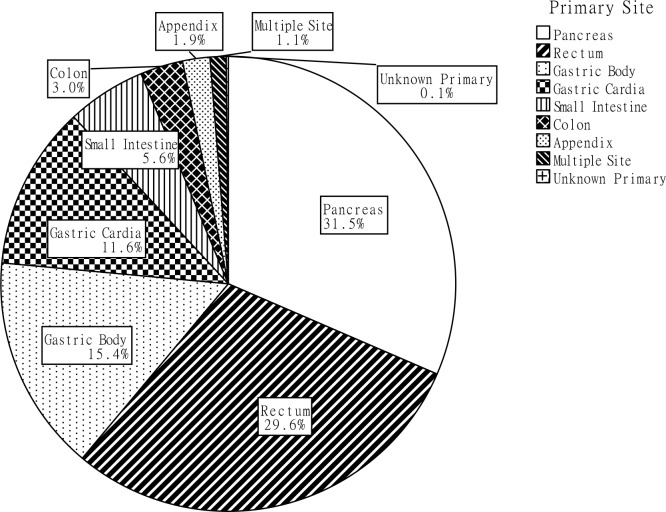
The proportion of primary tumor site in all cases

**Figure 2:**
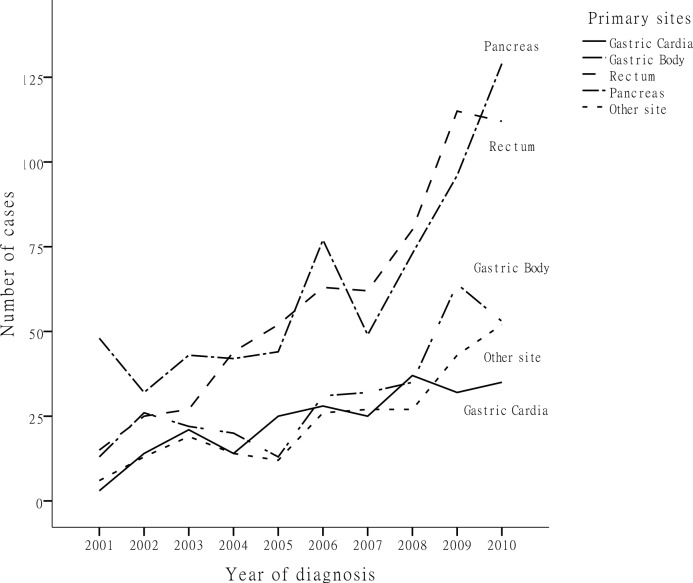
Number of patients diagnosed in each year

Of the 2,010 GEP-NEN cases, pancreatic NENs took up the majority, at 31.5% (633 cases), followed by rectal NENs at 29.6% (595 cases), gastric body NENs at 15.4% (309 cases) and cardiac NENs at 11.6% (234 cases). NENs originating at other sites, including the small intestine, colon, appendix, as well as multiple endocrine neoplasms, accounted for a relatively small proportion (Figure 1). Figure 2 shows the changes in GEP-NEN case number in each group over 10 years. Patient numbers in all sites increased consistently, with the highest increase in rectal and pancreatic NENs. Further analysis shows that the case number increased more significantly in urban than in rural residents, especially after the year 2006. We also compare the trend of case increase with Chinese cancer incidence in 2001-2010 [14, 15] (Supplementary Figure 1), and they showed similar changes during 2001-2009 (the different reference group result in the significant high cancer incidence in 2010). Considering the limited number of cases, we put NENs from the small intestine, colon, appendix, multiple and unknown sites in a single group. Table 1 shows the distribution of demographic characteristics and possible risk factors in patients of each primary site. Pancreatic and rectal NEN patients were diagnosed at a younger age. Male patients comprised of a larger proportion of all cardiac, gastric body and rectal NEN patients, at 84.2%, 74.1% and 57.3%, respectively, but female patients comprised of a larger proportion (58.8%) of pancreatic NENs. In rectal and pancreatic NEN patients, urban residents contributed over twice as many cases as rural residents. This situation was reversed in cardiac NENs, while gastric body NENs showed a more even distribution. A higher education level was more commonly seen in rectal and pancreatic NEN patients than in cardiac and gastric body NEN patients. Family history of tumors seemed to increase risk more for cardiac and gastric body NEN patients than for rectal and pancreatic NEN patients. The distribution of smoking and alcohol use significantly differed between the five groups (*P*<0.001).

**Table 1:** Basic characteristics and potential risk factors of GEP-NENs patients

Variables	Total distribution (N=2010)	Gastric cardiac NENs (N=234)	Gastric body NENs (N=309)	Rectal NENs (N=595)	Pancreatic NENs (N=633)	Other NENs (N=239)	*P* value
	n (%)	n (%)	n (%)	n (%)	n (%)	n (%)	
**Age at Diagnosis (M, QR)**	53.0, 20.0	61.0, 12.3	58.0, 17.5	51.0, 18.0	46.0, 20.0	53.0, 22.0	0.000*
**Sex**	**2010 (100.0)**	**234 (100.0)**	**309 (100.0)**	**595 (100.0)**	**633 (100.0)**	**239 (100.0)**	
Male	1169 (58.2)	197 (84.2)	229 (74.1)	341 (57.3)	261 (41.2)	141 (59.0)	0.000*
Female	841 (41.8)	37 (15.8)	80 (25.9)	254 (42.7)	372 (58.8)	98 (41.0)	
**Region**	**1909 (100.0)**	**227 (100.0)**	**291 (100.0)**	**566 (100.0)**	**596 (100.0)**	**229 (100.0)**	
Rural	609 (31.9)	145 (63.9)	125 (43.0)	103 (18.2)	179 (30.0)	57 (24.9)	0.000*
Urban	1300 (68.1)	82 (36.1)	166 (57.0)	463 (81.8)	417 (70.0)	172 (75.1)	
**Education level**	**503 (100.0)**	**31 (100.0)**	**64 (100.0)**	**202 (100.0)**	**122 (100.0)**	**84 (100.0)**	
Below middle school	178 (35.4)	15 (48.4)	30 (46.9)	70 (34.7)	32 (26.2)	31 (36.9)	0.032*
Middle school or above	325 (64.6)	16 (51.6)	34 (53.1)	132 (65.3)	90 (73.8)	53 (63.1)	
**Body Mass Index**	**1132 (100.0)**	**158 (100.0)**	**175 (100.0)**	**286 (100.0)**	**399 (100.0)**	**114 (100.0)**	
Underweight (≤18.49)	78 (6.9)	11 (7.0)	14 (8.0)	25 (8.7)	14 (3.5)	14 (12.3)	0.000*
Normal Weight (18.50-24.99)	510 (45.1)	73 (46.2)	93 (53.1)	125 (43.7)	157 (39.3)	62 (54.4)	
Overweight (25.00-29.99)	388 (34.3)	52 (32.9)	61 (34.9)	112 (39.2)	129 (32.3)	34 (29.8)	
Obese (≥30.00)	156 (13.8)	22 (13.9)	7 (4.0)	24 (8.4)	99 (24.8)	4 (3.5)	
**Tumor Family History**	**1911 (100.0)**	**226 (100.0)**	**300 (100.0)**	**548 (100.0)**	**613 (100.0)**	**224 (100.0)**	
Yes	81 (4.2)	12 (5.3)	22 (7.3)	18 (3.3)	21 (3.4)	8 (3.6)	0.037*
No	1830 (95.8)	214 (94.7)	278 (92.7)	530 (96.7)	592 (96.6)	216 (96.4)	
**Smoking Status**	**1844 (100.0)**	**233 (100.0)**	**288 (100.0)**	**507 (100.0)**	**592 (100.0)**	**224 (100.0)**	
Smoker	533 (28.9)	106 (45.5)	114 (39.6)	139 (27.4)	114 (19.3)	60 (26.8)	0.000*
Non-smoker	1311 (71.1)	127 (54.5)	174 (60.4)	368 (72.6)	478 (80.7)	164 (73.2)	
**Alcohol Drinking Status**	**1833 (100.0)**	**232 (100.0)**	**285 (100.0)**	**504 (100.0)**	**590 (100.0)**	**222 (100.0)**	
Drinker	427 (23.3)	73 (31.5)	80 (28.1)	125 (24.8)	99 (16.8)	50 (22.5)	0.000*
Non-drinker	1406 (76.7)	159 (68.5)	205 (71.9)	379 (75.2)	491 (83.2)	172 (77.5)	

* Significantly different, using Kruskal-Wallis test, Mantel-Haenszel χ^2^ test or Fisher's exact tests.

### Neoplasm stage and histological grade

**Figure 3:**
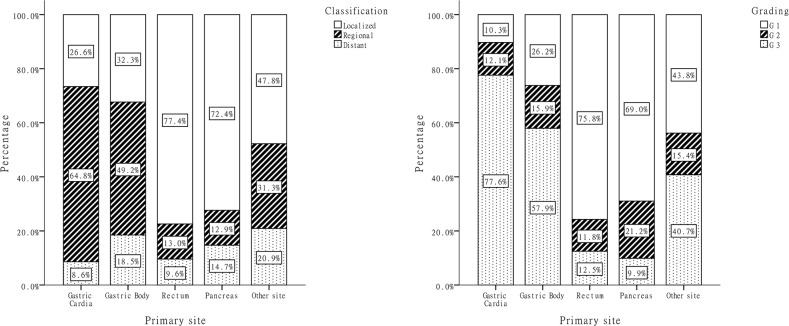
Distribution of classification and grade by primary anatomic sites

We received classification information for 1,903 cases, and 4.1% (81 cases) cases could not be classified due to insufficient information. Grading based on mitosis rate or the Ki-67 labeling index was explicit in 1,456 (73.4%) cases. The stage and grade distribution in each site group were shown in Figure 3. Tumor stage (*P*<0.001) and grade (*P*<0.001) varied greatly based on primary tumor site: 77.4% of rectal and 72.4% of pancreatic NENs were diagnosed at the localized stage and at grades G1/G2 (87.6% and 90.2%), while more cardiac and gastric body NENs (73.4% and 67.7%) displayed local invasion or distant metastasis at diagnosis. Among the 1,414 cases where both stage and grade information were available, we found that histological grade was strongly associated with disease stage (*P*<0.001): 8.7% (of 732) of G1 and 16.9% (of 219) of G2 NEN patients exhibited synchronous distant metastasis at diagnosis, whereas 17.7% (of 463) of G3 NEN patients had metastasis at diagnosis.

### Treatment methods

Overall, 1,820 (90.5%) of the GEP-NEN patients underwent surgery, of which more than 90% were curative and the rest were palliative. Palliative surgery was performed most frequently (9.8% of all surgeries) in rectal NEN patients. Over 90% of the surgeries were performed as open surgeries for cardiac, gastric body and pancreatic NEN patients, and surgeries under endoscopy were performed most frequently on rectal NENs (28.4%). A total of 393 (20.0%) of 1,967 patients have underwent chemotherapy, separately or before surgery, with higher rates among cardiac and gastric body NEN patients. Biotherapy was given to 171 (27.5%) of 621 pancreatic NEN patients but was uncommon for patients with other primary NEN sites. Other therapies, such as targeted therapy and radiotherapy were rarely used (Table 2).

**Table 2:** Surgery and other therapy information of NENs from different anatomic sites

Treatment method	Total distribution (N=2010)	Gastric cardiac NENs (N=234)	Gastric body NENs (N=309)	Rectal NENs (N=595)	Pancreatic NENs (N=633)	Other NENs (N=239)	*P* value
	n (%)	n (%)	n (%)	n (%)	n (%)	n (%)	
**Surgery type**	**1820 (100.0)**	**225 (100.0)**	**265 (100.0)**	**533 (100.0)**	**585 (100.0)**	**212 (100.0)**	
Radical surgery	1699 (93.4)	221 (98.2)	246 (92.8)	481 (90.2)	562 (96.1)	189 (89.2)	0.000*
Palliative surgery	121 (6.6)	4 (1.8)	19 (7.2)	52 (9.8)	23 (3.9)	23 (10.8)	
**Surgery method**	**1699 (100.0)**	**204 (100.0)**	**265 (100.0)**	**464 (100.0)**	**555 (100.0)**	**211 (100.0)**	
Open surgery	1446 (85.1)	199 (97.5)	241 (90.9)	309 (66.6)	507 (91.4)	190 (90.0)	0.000*
Laparoscopic surgery	107 (6.3)	5 (2.5)	19 (7.2)	23 (5.0)	47 (8.5)	13 (6.2)	
Endoscopic surgery	146 (8.6)	0 (0)	5 (1.9)	132 (28.4)	1 (0.2)	8 (3.8)	
**Chemotherapy**	**1967 (100.0)**	**224 (100.0)**	**304 (100.0)**	**579 (100.0)**	**627 (100.0)**	**233 (100.0)**	
Yes	393 (20.0)	82 (36.6)	115 (37.8)	72 (12.4)	69 (11.0)	55 (23.6)	0.000*
No	1574 (80.0)	142 (63.4)	189 (62.2)	507 (87.6)	558 (89.0)	178 (76.4)	
**Biotherapy**	**1982 (100.0)**	**231 (100.0)**	**307 (100.0)**	**589 (100.0)**	**621 (100.0)**	**234 (100.0)**	
Yes	195 (9.8)	0 (0)	6 (2.0)	3 (0.5)	171 (27.5)	15 (6.4)	0.000*
No	1787 (90.2)	231 (100.0)	301 (98.0)	586 (99.5)	450 (72.5)	219 (93.6)	
**Target therapy**	**1980 (100.0)**	**226 (100.0)**	**305 (100.0)**	**585 (100.0)**	**629 (100.0)**	**235 (100.0)**	
Yes	14 (0.7)	0 (0)	6 (2.0)	0 (0)	6 (1.0)	2 (0.9)	0.004*
No	1966 (99.3)	226 (100.0)	299 (98.0)	585 (100.0)	623 (99.0)	233 (99.1)	
**Radiotherapy**	**1997 (100.0)**	**231 (100.0)**	**308 (100.0)**	**590 (100.0)**	**632 (100.0)**	**236 (100.0)**	
Yes	43 (2.2)	7 (3.0)	5 (1.6)	20 (3.4)	5 (0.8)	6 (2.5)	0.024*
No	1954 (97.8)	224 (97.0)	303 (98.4)	570 (96.6)	627 (99.2)	230 (97.5)	

* Significantly different, using Mantel-Haenszel χ^2^ test or Fisher's exact tests.

### Clinical and pathologic characteristics

Dyspepsia was the most common presenting symptom for patients with gastrointestinal neoplasms, and was seen in 12.4% of 1336 patients with valid data. Among patients with pancreatic NENs, 47.5% presented with Whipple triad symptoms, which were the most common hormone-related symptoms overall. Diarrhea was recorded in 139 cases. Most of them were in rectal NENs (75 cases), which comprised 12.6% of all rectum NEN patients. Flush and Zollinger-Ellison syndromes were respectively shown in 15 and 20 GEP-NEN cases. Verner-Morrison syndrome and glucagonoma syndrome was presented in 5 and 12 Pan-NEN patients, respectively. As shown in Table 3, rates of vascular space involvement, perineural invasion, and necrosis were higher in cardiac, gastric body and pancreatic NENs, while cystic degeneration was more likely found in pancreatic NENs; rates of all of these differed according to the NEN sites (P<0.001). Among patients with biomarker test information, about 90% patients were positive for synaptophysin positive, a much higher rate than choromogranin A, CD56, neuron-specific enolase, S-100 protein, or CK (AE1/AE3).

**Table 3:** Pathological features and immunohistochemistry results of certain biomarkers of NENs

Clinical and Pathological features	Total distribution (N=2010)	Gastric cardiac NENs (N=234)	Gastric body NENs (N=309)	Rectal NENs (N=595)	Pancreatic NENs (N=633)	Other NENs (N=239)	*P* value
	n (Positive rate %)	n (Positive rate %)	n (Positive rate %)	n (Positive rate %)	n (Positive rate %)	n (Positive rate %)	
**Vascular space involvement**	1390 (13.8)	209 (22.0)	252 (19.0)	497 (5.2)	258 (17.4)	174 (15.5)	0.000*
**Perineural invasion**	1378 (5.2)	207 (5.8)	250 (6.8)	493 (1.8)	256 (9.0)	172 (6.4)	0.000*
**Necrosis**	1391 (9.1)	208 (13.9)	249 (13.3)	499 (3.8)	266 (11.3)	169 (9.5)	0.000*
**Cystic degeneration**	1368 (2.6)	204 (1.5)	241 (0.4)	490 (0.8)	266 (9.8)	167 (1.2)	0.000*
**Synaptophysin**	1296 (90.0)	168 (91.1)	215 (89.3)	410 (88.8)	327 (93.3)	176 (86.9)	0.149
**Choromogranin A**	1243 (67.6)	148 (62.8)	192 (75.0)	371 (52.3)	376 (81.6)	156 (65.4)	0.000*
**CD56**	532 (79.7)	94 (80.9)	111 (75.7)	143 (77.6)	117 (89.7)	67 (71.6)	0.021*
**Neuron-specific enolase**	612 (78.8)	78 (65.4)	99 (67.7)	218 (85.3)	135 (83.7)	82 (79.3)	0.000*
**S-100 protein**	205 (41.0)	26 (26.9)	33 (45.5)	64 (48.8)	49 (34.7)	33 (42.4)	0.32
**CK(AE1/AE3)**	940 (79.1)	122 (82.0)	165 (86.1)	300 (82.7)	227 (70.5)	126 (74.6)	0.001*

* Significantly different, using Mantel-Haenszel χ^2^ test or Fisher's exact tests.

## DISCUSSION

Previous studies in China have revealed the clinical features of NENs in single center [16, 17], but this was the first geographically representative epidemiologic study for GEP-NEN. In this study, we reviewed information from 2,010 patients and assessed the general epidemiological characteristics, clinical symptoms and treatment information of GEP-NENs.

The most common GEP-NENs began in the pancreas, rectum and the stomach, each comprising almost 30% of all NENs, if we put the gastric cardiac and gastric body together. Small intestinal, colonic, and appendicular NENs were relatively rarely seen. Our findings significantly differed from SEER [9]. According to SEER 17 (2000-2007), the rectum and small intestine were the most common sites for NENs (29.2% and 28.4% of all GEP-NENs); followed by those in the pancreas and colon (11.5% each), and finally those in the stomach and appendix were the least common (9.9% and 5.1%). Studies based on databases from Norway [18] and Switzerland [19] also revealed a high incidence of small intestinal and colorectal NENs, while the incidence of appendix NENs surpassed that of small intestinal and colorectal NENs in the UK [10]. These inconsistencies were due in part to racial disparities; Yao [8] reported that among Asians/Pacific Islanders in American, the incidence of rectal NENs patients was about 5 times that of small intestinal, pancreatic and gastric NENs, but these rates were not observed in Caucasians or African Americans. Studies in Korea [11] and Taiwan [13] found similar results to those of Asians/Pacific Islanders in America, reporting that incidence of rectal NENs was almost 3-4 times that of gastric, pancreatic, colon and small intestinal. Results from a Japanese study also found that midgut NENs accounted for only a small percentage of all NENs [12]. In addition, as the small intestinal NENs are always asymptomatic, and only by metastasis to the liver, watery diarrhea and flushing would be shown. Also, Small intestine is an organ which is hard to be accessed, and the tumor diagnose rate is more dependent to endoscopic procedures comparing to tumors of other sites. The low detection rate of small intestinal NEN in our study was also partly due to the inferior radiographic and endoscopic techniques. We found that NENs of the upper digestive tract were more common in rural residents, while NENs of the rectum and pancreas were more common among urban residents. According to the annual cancer reports in China from 2007-2010 [15], the incidence of gastric neoplasms was over one-fold higher in rural than that among urban residents, while colorectal neoplasms had almost 1.5-fold higher incidence in urban residents. As denocarcinoma and NENs from the same sites may share similar risk factors, the different predilection part of digestive tract may also have something to do with the nutrition level as some papers reported before [20]. Also, relating screening programs on upper digestive tumors were more frequently in rural than in urban could be another reason. The high incidence of upper digestive tract NEN incidence in rural area could partially explain the high incidence of gastric NENs in mainland China. On the other hand, The NEN patients of all sites were increasing during the past decade, and the better acknowledgment of doctors and diagnose capacity may be the main reasons. Pancreatic and rectum NENs were more likely to be symptomatic, and the case number increased more significantly. The remarkable case number increase in urban patients revealed the unequal development of medical recourses.

The diagnosis age for Chinese GEP-NEN patients was about 10 years younger than that of Americans (53.0 years vs. 62.0 years) [8] for all primary sites, especially for pancreatic NENs (45.7 years VS. 59.0 years). We also found that sex ratios of different anatomic sites varied greatly: male patients comprised most cardiac and gastric body NEN patients and a smaller majority in rectal NEN patients; female patients dominated in pancreatic NENs. Similar but less extreme sex ratios were reported in the Taiwanese study [13]. In western countries and American [8, 21], gastric NENs occurred equally in males and females, while males continued to dominate in rectal and pancreatic NENs.

Large disparities were also found between Asia and America [8] in terms of clinical stage and histology grade; almost 70% of gastric NENs in our study were at the regional or distant stage, while this proportion was only 30% in America. Rectal NENs were also diagnosed at later stage in our study; the proportion of NENs in the local stage was 72.4% vs. 92% in America. Most pancreatic NENs were benign, local, and graded as G1 when diagnosed in our study and in Japan [12], while in America, 64% of patients were at the distant metastasis stage [8].

It has been reported that surgery is the only potentially curative therapy for GEP-NENs [22–24], chemotherapy is effective for poorly-differentiated NENs [25–27], and biological therapy proved to be effective in some studies for reducing hormone-related symptoms [28, 29]. In our study, surgery was the most common therapy; chemotherapy was used frequently for cardiac and gastric body NENs, while biological therapy was only used in some patients with pancreatic NENs. Other therapeutic options, such as targeted therapy and radiotherapy, were rarely used in China. The distribution of most prognosis related [30–32] pathological features and biomarker detection rates differed according to anatomic site in our study too.

These findings must be considered in the light of the study's strengths and weaknesses. Firstly, as a multi-center study, the case number was large and the hospitals were geographically represented. Secondly, the variables we collected covered parameters of epidemiology, clinical presentation, diagnosis, and pathology, providing a fairly complete profile of the GEP-NEN patients. Thirdly, we used basic indicators and the newest classification standard to reduce information bias. The main limitations were: first, the hospitals included were convenience-sampled, and they were more likely to be in advanced medical level. Some early-stage lesions removed surgically in local hospitals may be missed. Secondly, we have no comparison group to evaluate risk factors. Finally, data quality depended partially on the integrity of the medical records.

In summary, we found that in Chinese tertiary hospitals, pancreatic and rectal NENs comprised the majority of all GEP-NENs, and the sex, age, stage and grade were various depending on the primary neoplasm sites. Multiple examination and treatment were limited in the current clinical course. Our results provide primary data on GEP-NEN patients in China, and suggest the different epidemiology and clinical features of GEP-NENs in China and other countries. Additional observation studies with more superior sampling methods are needed to confirm these findings.

## MATERIALS AND METHODS

### Study design

This study was a hospital-based, multi-center, retrospective study over 10 years (2001-2010). All GEP-NEN patients with confirmed pathology from the selected hospitals were included for analysis.

### Selection of hospitals and patients

China was divided into seven geographical regions based on traditional administration: North, North-East, Central, South, East, North-West and South-West. At least one cancer hospital and one general hospital were selected from each region. Convenience sampling was used to choose hospitals from each region. The inclusion criteria for hospitals included: (1) at tertiary level which cover patients from different parts of the region; (2) easy retrieval of information on GEP-NEN patients and (3) a local team with the ability to complete the case report form. The inclusion criteria for patients included (1) pathological diagnosed as GEP-NEN patients in the years 2001-2010; and (2) possessing complete medical records. All eligible records from the selected hospitals were reviewed based on the designed case report form (CRF). This study was approved by the Institutional Review Board of the Cancer Hospital, Chinese Academy of Medical Sciences.

### Data collection and quality control

The case report form was designed by professionals of epidemiology, pathology, internal medicine, surgery, imaging, and radiotherapy, and the pilot study was conducted in CHCAMS. The questions in case report form included eight aspects: (1) general administrative information; (2) demographic characteristics and possible risk factors at the time of diagnosis, including age, gender, height and weight, occupation, education and marital status, household registration, family history of tumor (whether malignant or benign, occurred on parents or siblings), smoking and drinking status (have the habits of smoking or drinking now, whether how long the history is); (3) linical features, including primary tumor sites and clinical syndromes; (4) results of imaging tests, including transabdominal ultrasound, computed tomography, endoscopy and ultrasound endoscopy; (5) use of currently available treatment approaches, including surgery, chemotherapy, biological therapy and target therapy; (6) pathological characteristics, including tumor size, infiltration limits, mitosis rate, Ki-67 labeling index, vascular space involvement; (7) immunohistochemical results of biomarker tests, including synaptophysin, choromogranin A, and neuron-specific enolase.

All information was abstracted from the medical records by a trained local doctor. Two copies of data set entered by two clerks via EpiData were sent to the study center in CHCAMS. Consistency validation and logistical checks were carried out and the final database was used for analysis. De-identified data were stored in a security database (FoxPro) via a recognizable series of index numbers. There data were accessible only to researchers and will be reported in aggregate.

### Data processing

The staging system applied to NENs during the last decade was not unified [33, 34], so we used the basic information (including tumor site, size, lymph node involvement, invasion range, and metastatic status) that determines tumor staging to reclassify the 2,010 cases as localized, regional, or distant. Localized NENs are confined entirely to the original organ; regional NENs meet at least one of the following conditions: (1) the tumors invaded surrounding organs or tissues or (2) the tumors involved regional lymph nodes; and distant NENs spread to other body parts remote from the primary tumor site. Tumor grade was determines by Ki-67 index or mitotic count, if both results were available, the higher one was taken.

### Data analysis

Patients’ age was tested for normality using the one-sample Kolmogorov-Smirnov test, and the Kruskal-Wallis test were used to compare the median of each group. Discrete variables were examined using Mantel-Haenszel chi-square tests and Fisher's exact tests to obtain *P* values. SPSS statistical software version 17.0 (SPSS Inc. Chicago, IL, USA) was used to analyze the data. Statistical significance was assessed by two-tailed tests with α level of 0.05.

## SUPPLEMENTARY FIGURE



## Abbreviations

GEP-NEN: Gastroenteropancreatic Neuroendocrine Neoplasm
WHO: World Health Organization
ENETS: the European Neuroendocrine Tumor Society
AJCC/UICC: the American Joint International Cancer/ Union of International Cancer
SEER: the Surveillance
CRF: Case Report Form
