# Supplementary material for: A nation-wide retrospective epidemiological study of gastroenteropancreatic neuroendocrine neoplasms in china
Source: Oncotarget. 2017 May 3;8(42):71699–708. doi: 10.18632/oncotarget.17599 (PMC5641082; doi:10.18632/oncotarget.17599)
Supplement: Supplementary file 1 [file oncotarget-08-71699-s001.pdf]

A nation-wide retrospective epidemiological study of gastroenteropancreatic neuroendocrine neoplasms in china

SUPPLEMENTARY MATERIALS

SUPPLEMENTARY FIGURE

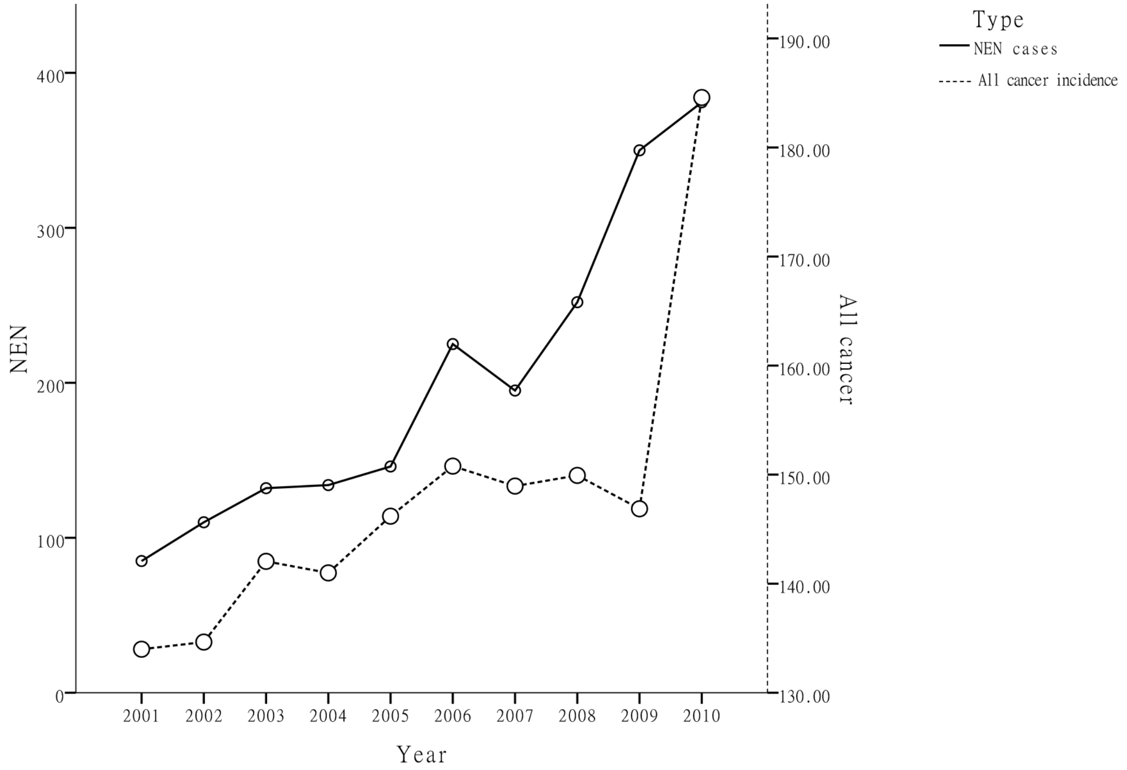

Supplementary Figure 1: GEP-NEN case increase comparing with Chinese cancer incidence in 2001-2010.
